# Exploring factors influencing quality of life variability among individuals with coeliac disease: an online survey

**DOI:** 10.1136/bmjgast-2024-001395

**Published:** 2024-06-02

**Authors:** Martha Elwenspoek, Jonathan Banks, Prajakta Pratap Desale, Jessica Watson, Penny Whiting

**Affiliations:** 1 University of Bristol Population Health Sciences, Bristol, UK; 2 NIHR ARC West, Bristol, UK

**Keywords:** COELIAC DISEASE, QUALITY OF LIFE, GLUTEN FREE DIET

## Abstract

**Objective:**

Patients with coeliac disease (CD) need to follow a strict gluten-free diet to manage symptoms and prevent complications. Restrictions imposed by the diet can be challenging and affect quality of life (QoL). We explored sources of variation in QoL among patients with CD.

**Design:**

We conducted an online survey of coeliac patients in the UK, including a CD-specific QoL tool (CD-QOL V.1.0), questions on diet adherence and an optional comment box at the end. The survey was disseminated via social media and went live between January and March 2021. We performed multiple linear regression and free text analysis.

**Results:**

We found a median CD-QOL score of 61 (IQR 44–76, range 4–100, n=215) suggesting good QoL (Good >59); however, the individual QoL scores varied significantly. Regression analyses showed that people who found diet adherence difficult and people adhering very strictly had a lower QoL. Free text comments suggested that people who adhered very strictly may do so because they have symptoms with minimal gluten exposure. People who found diet adherence difficult may be people who only recently started the diet and were still adjusting to its impact. Comments also highlighted that individuals with CD often perceive a lack of adequate follow-up care and support after diagnosis.

**Conclusion:**

Better support and follow-up care is needed for people with CD to help them adjust to a gluten-free diet and minimise the impact on their QoL. Better education and increased awareness are needed among food businesses regarding cross-contamination to reduce anxiety and accidental gluten exposure.

**WHAT IS ALREADY KNOWN ON THIS TOPIC**

- After diagnosis and start of treatment, quality of life in people with coeliac disease normalises in some but not in others. To better support patients with coeliac disease, it is essential to understand the factors that cause this variability.

**WHAT THIS STUDY ADDS**

- A lack of postdiagnosis support and awareness of coeliac disease make adhering to the diet unnecessarily challenging, which can affect quality of life. Our observations highlight inequalities in access to follow-up care in the UK.

**HOW THIS STUDY MIGHT AFFECT RESEARCH, PRACTICE OR POLICY**

- Quality of life of coeliac patients can be improved by ensuring equitable healthcare access across the UK, including gluten-free prescriptions, access to a dietician and follow-up care irrespective of postcode, and by educating healthcare professionals to follow the existing guidance on postdiagnosis support.

## Background

Coeliac disease (CD) is a chronic autoimmune disorder that causes intestinal damage in response to consuming gluten-containing products.^1 2^ The disease is common with an estimated global prevalence of 1%.^3^ Clinical presentations vary and if left untreated CD can lead to serious complications, such as osteoporosis, anaemia, malnutrition, malignancies and premature death.^2 4–7^ CD cannot be cured, although symptoms can be managed effectively for most patients by following a gluten-free diet (GFD). Strict adherence to the diet is essential to prevent symptoms and reverse intestinal damage to prevent long-term complications.^4 8^

Research has shown that living with CD can have a negative impact on quality of life (QoL).^9^ People with undiagnosed CD have a substantially lower QoL than the general population, mainly due to the impact of symptoms in daily life.^10 11^ Symptoms can improve within days to weeks on a GFD.^12^ Once diagnosed and treatment is started, QoL improves^10 13–15^ but may not normalise.^16^

The diet is restrictive, and it can be difficult for people to adjust to. It requires lifestyle changes and a great deal of knowledge about food labels to recognise gluten-containing products. People who follow a GFD can no longer eat foods that are considered everyday items such as bread, pasta, biscuits, pastries and soups unless there are special gluten-free versions. They also need to check processed food labels for additives that contain gluten. Coeliac patients need to be careful of cross-contamination, which poses a risk at every stage of food processing. A total of 2.1%–37% of labelled and unlabelled gluten‐free products are affected by cross-contamination.^14^

The goal is to eliminate gluten from the diet as much as possible because complete elimination is not achievable in practice. Recent studies report a high rate of gluten exposure even among patients with apparently strict adherence.^17–19^ Therefore, regular follow‐up to monitor diet adherence based on history and coeliac serology is recommended as well as support by a dietician to identify possible remaining sources of gluten.^1 4 20^

We conducted a survey of coeliac patients to look at QoL and sources of variation in QoL among patients with CD.

## Methods

### Survey design and dissemination

This cross-sectional study used a subgroup of respondents to a wider survey conducted in 2021. Survey questions were developed in collaboration with GPs, gastroenterologists and patient representatives on the project team after which revisions were made based on input from a focus group of four patient representatives and a plain-language panel. The aim of the wider survey was to assess how confident people want to be in their diagnosis before starting a GFD or undergoing a biopsy and included people with and without CD (n=472).^9^ Adults living in the UK with CD that was confirmed by a clinician and/or a test (serology or biopsy/endoscopy) who completed the CD-QOL questionnaire were included in this analysis. Data were collected online between January and March 2021. It was disseminated via Facebook, citizen panels, Twitter and Coeliac UK to reach a diverse group of people. Informed consent was obtained by agreeing to participate in the survey. Development and dissemination of the full survey are described in detail here.^9^

### Survey questions

The CD-QOL V.1.0 questionnaire, developed in 2010,^21^ consists of 21 questions: 20 questions use a 5-point Likert scale with 1 being ‘Not at all’ and 5 being ‘A great deal’ covering four clinical aspects: dysphoria (four questions), CD-related restrictions (nine questions), health concerns (five questions) and inadequate treatment (two questions); and the final question asks participants to self-rate their QoL on a 5-point Likert scale with 1 being ‘Excellent’ and 5 being ‘Poor’.^17^ We also asked respondents how strictly they adhered to a GFD and how difficult they found adhering to it. We collected sociodemographic data, including age, sex, educational qualification and area of residence. Respondents could add any comments in an optional free text field at the end of the survey.

### Data analysis

Data cleaning steps in the original sample are described here.^9^ In brief, ethnicity was summarised with a binary variable. Qualifications were recoded, keeping only the highest qualification. Postcodes were used to link to deprivation deciles. In case of a missing value, an average score of the other items in the entire questionnaire was assigned to that section. Raw scores were calculated for overall QoL and four clinical domains. These raw scores were then used to calculate the total QoL score and individual total scores for each domain using the following formula: (((raw score)−(number of questions))/(number of questions)×4)×100). Based on the total scores, the QoL was categorised as good (>59), medium (37–59) or poor (<37) as per the developer’s instructions. Missing data in the CD-QOL questions were imputed by using the average (median) score of the relevant domain. We made the assumption that people missed a question by accident, and, therefore, the missing answers were missing at random.

Descriptive statistics were calculated to describe population characteristics using relative frequencies (in %) for categorical variables and median, IQR and ranges for numerical outcomes.

We performed an exploratory analysis to identify possible causes of variation in QoL. We performed multiple linear regression to investigate whether diet adherence and experienced difficulty of the diet were associated with QoL. We accounted for age group and sex in the model.

We analysed the free text by categorising based on the themes derived from the QoL questionnaire to link it back to the quantitative results for each QoL domain using NVivo V.1.7.1 software. Subthemes were identified inductively from the data by ME and checked by JB. Quotes were labelled with multiple categories if they matched with more than one category. We then categorised quotes as positive or negative in relation to their potential impact on QoL.

## Results

### Study sample characteristics

The subsample selected for this analysis included a total of 215 adults living in the UK with CD confirmed by a test (serology and/or biopsy) or clinician (see online supplemental figure 1 for a participant flowchart). The majority of respondents were aged between 26 and 64 years (n=158, 75.5%). Questions on sex, ethnicity, qualifications and postcode were optional but were answered by most people. The vast majority of respondents were white (n = 207, 96.3%) and female (n = 195, 90.7%). Most respondents went to university or college (n=130, 60.5%). The highest proportion lived in Southwest England (n=70, 32.6%). Respondents tended to live in less deprived areas (median deprivation index of 7, IQR 5–8) than the national average (table 1).

10.1136/bmjgast-2024-001395.supp1 Supplementary data

**Table 1:** Study sample characteristics

		Total (N=215)
Sex	Women	195 (90.7%)
	Man	16 (7.4%)
	Other/missing	4 (1.9%)
Age (years)	18–25	31 (14.4%)
	26–40	66 (30.7%)
	41–64	92 (42.8%)
	65+	26 (12.1%)
Ethnicity	White	207 (96.3%)
	Non-white	7 (3.3%)
	Missing	1 (0.5%)
Highest education	College or university degree	130 (60.5%)
	A levels or equivalent	37 (17.2%)
	O levels or GCSEs or equivalent	27 (12.6%)
	Other/missing	21 (9.8%)
Deprivation score	1–2	17 (7.9%)
	3–4	28 (13.0%)
	5–6	38 (17.7%)
	7–8	56 (26.0%)
	9–10	42 (19.5%)
	Missing	34 (15.8%)
Region	East England	17 (7.9%)
	East Midlands	13 (6.0%)
	Greater London	10 (4.7%)
	North East	16 (7.4%)
	North West	20 (9.3%)
	Northern Ireland	4 (1.9%)
	Scotland	12 (5.6%)
	South East	25 (11.6%)
	South West	70 (32.6%)
	Wales	8 (3.7%)
	West Midlands	13 (6.0%)
	Missing	7 (3.3%)

GCSEs, General Certificate of Secondary Education.

### Gluten-free diet

Of 99.1% respondents followed a GFD and the majority followed the diet strictly (n=148, 68.8%) or very strictly (n=45, 20.9%; online supplemental table S1). One respondent never followed a GFD, one did so in the past, and one tries to lower their intake but is still eating gluten-containing products. There was much heterogeneity in how difficult people found adhering to the GFD. Almost as many people found it very easy (40%) as difficult (55%).

### QoL scores and self-reported QoL

**Figure 1:**
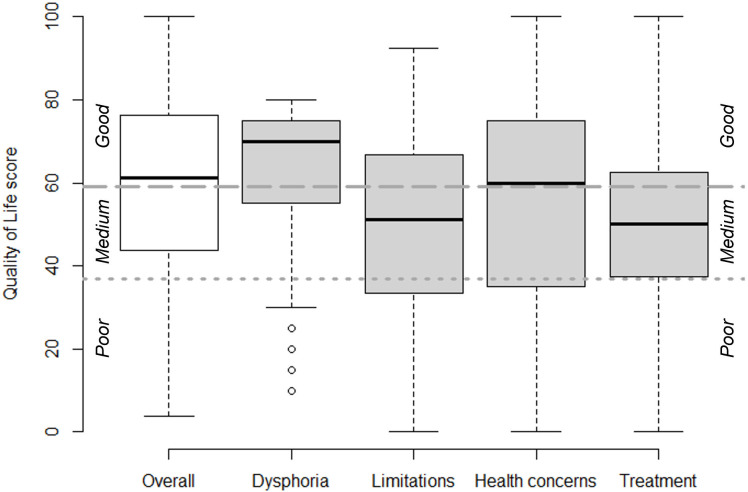
Quality of life score (CD-QOL). The leftmost boxplot (white) represents the overall quality of life score. The next four boxes (grey) represent the separate domains of quality of life. The line in the middle of the box represents the median. The outer edges of the box represent the IQR, and the whiskers represent the range. Single dots represent outliers. The dotted grey line depicts the 37 CD-QOL score cut-off between poor and medium quality of life, and the striped grey line depicts the 59 CD-QOL score cut-off between medium and good quality of life.

The median overall CD-QOL score was estimated at 61 (IQR 44–76, range 4–100). This suggests that just over half of the respondents had a good QoL (CD-QOL score >59), and the remaining had a medium (CD-QOL score 37–59) or poor QoL (figure 1). Respondents had a high score in the dysphoria domain (median 70, IQR 55–57, range 10–80), suggesting that dysphoria did not affect QoL for the majority of respondents. The remaining domains showed more variation. For the CD-related restrictions and inadequate treatment domains, the majority of respondents scored a medium or poor QoL (median 51, IQR 33–67, range 0–92; median 50, IQR 38–63, range 0–100, respectively). Similarly, the health concern domain showed substantial heterogeneity, but with on average a ‘good’ QoL (median 60, IQR 35–75, range 0–100).

The average self-rated QoL was ‘Good’ (‘How would you rate your QoL related to your illness?’); although, consistent with the calculated scores, there was substantial variation. A small proportion rated their QoL ‘Poor’ (2.8%, online supplemental table S2). Although we found strong evidence of a correlation between self-rated QoL and CD-QOL scores (r=0.61, 95% CI 0.52 to 0.69, p<0.001), the correlation itself was relatively weak.

### QoL scores and GFD adherence

**Figure 2:**
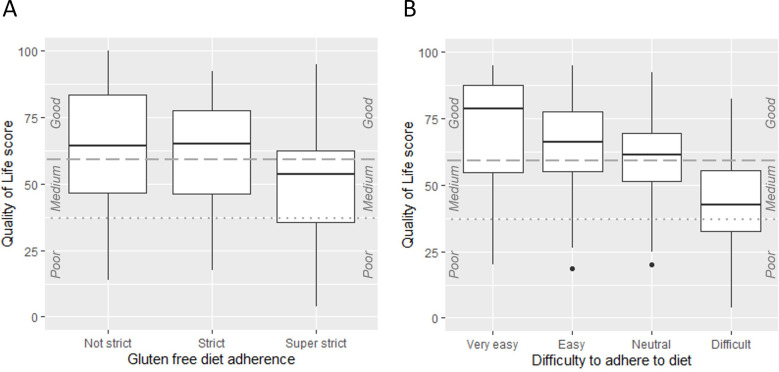
Quality of life versus gluten-free diet adherence (A) and difficulty adhering to the gluten-free diet (B). (A) The ‘Not strict’ group includes people who answered ‘Not at all’, ‘Not very strict’ and ‘Relatively strict’. (B) The ‘Difficult’ group includes people who answered ‘Difficult’ and ‘Very difficult’.

We found strong evidence of an association between QoL and how strictly people reported adhering to the diet as well as how difficult people found adhering to the diet. People who adhered very strictly to the diet had a significantly lower QoL than people who adhered ‘strict’ or ‘not strict’ (figure 2A). They scored 20 points lower on QoL compared with the ‘not strict’ group (SE 4.7, t=–4.4; p<0.001). We found a dose–response relationship between how difficult people found it to adhere to the diet and their QoL score (figure 2B). The more difficult people found it, the lower their QoL score. People who found the diet difficult or very difficult scored 28 points lower on QoL compared with people who found it very easy (SE 3.5, t=–8.0; p<0.001).

### Free text answers

A total of 143 people left a comment at the end of the survey, of which 98 included comments related to their QoL. Comments were related to all four domains of the CD-QOL questionnaire, but most comments (45 comments) were related to restrictions. We identified one additional theme and several subthemes within each domain. Table 2 shows example quotes per theme and subtheme (for all categorised comments, see online supplemental table S3).

**Table 2:** Themes and example quotes

Theme and subthemes	Example quotes
Dysphoria	
Experiencing the disease as a burden and feeling overwhelmed (13 comments)	*‘Having this disease is a heavy burden, the [gluten free] options are very expensive where they are available, choices are limited, you have to shop in 5 different supermarkets due to [the] exclusivity of brands. It runs your life having to check ingredient labels constantly, being limited to eating out with friends and family. Feel persecuted and victimised at times. It really upsets me when I can't be normal and included with everyone else*.’
Insufficient support and information (9 comments)	‘*I live in constant fear of being contaminated when I eat out because it has happened frequently to me even though I am so careful, and there’s not enough support for coeliacs dealing with this*.’
Social anxiety (10 comments)	‘*I feel an inconvenience to family occasions and celebrations & often opt not to eat rather than come across as problematic. This disease begins to affect your personality & your mental health as much as your physical health*.’
Health concerns	
Worries about other conditions linked to CD (10 comments)	*‘I have only been given the standard bone health help for elderly people and no tailored advice for someone young who is actively trying to BUILD bone. It’s just very hard to be happy when you're always worried about your health.’*
Health worries as a result of a late diagnosis (7 comments)	*‘It took me 22 years to be diagnosed from when symptoms started, and I worry about the damage that time has done. It also made it a lot harder to come to terms with.’*
Inadequate treatment	
Wish for better treatment options (4 comments)	*‘I really hope that there is a solution to cross contamination, and, if accidently occurs, then there should be a medicine to prevent damage.(…)Avoiding all gluten even at microscopic levels is very stressful, and it makes life a lot harder.’*
Positive attitude related to treatment	*‘I think the gluten-free diet is a very small price to pay for the benefits on the health of anyone with CD.’*
CD-related restrictions	
Lack of awareness or understanding of CD (31 comments)	*‘There is continual frustration with the common misconception that it is a lifestyle choice not a disease that can have horrid symptoms.’* *‘Was fobbed off so many times that my symptoms were because of a stressful job. Diagnosed with anaemia before CD, but no follow-up blood test to see the tablets weren’t being absorbed. I had to ask for a blood test.’*
Restrictions of gluten-free products (25 comments)	*‘I think there should be more financial support towards the cost of eating [gluten free] for coeliacs the cost of the food has impacted my shopping budget a lot. I worry the financial impact it would have if my 4 children also had the disease.’*
Restrictions on social life (12 comments)	*‘Food out and about is always tricky, and I know my family find it frustrating when we are on holiday etc, plus limited options.’*
Lack of follow-up	
Concern regarding follow-up care after diagnosis (10 comments)	*‘My follow-up on care has been lacking; I've been diagnosed within the last 6 months and have had a follow-up telephone appointment and then pretty much signed off. Told to speak to my GP in a year, no information about whether bloods are needed to confirm I'm on the right track etc.’*

#### Dysphoria

Three subthemes related to dysphoria emerged from the comments of respondents. First, experiencing the disease as a burden and feeling overwhelmed: respondents described having CD as a ‘heavy burden’, ‘very difficult’ and ‘incredibly overwhelming and impactful’, and it was mentioned that it negatively impacted their mental health. Second, receiving insufficient support and information: respondents felt that they did not know enough about the disease and did not receive enough information at diagnosis about what to expect or support adjusting to a gluten-free lifestyle. In contrast, one person mentioned that there is a lot of support on social media, and they felt well informed by online information. Third, social anxiety: respondents describe the struggle with social situations, being forced to be ‘difficult’ or ‘the fussy one’, which leads to feeling awkward and uncomfortable. Some people prefer to avoid these situations all together and miss out on social events. Eating out is a cause of anxiety for many because of social awkwardness and because of the fear of cross-contamination and resulting symptoms.

#### Health concerns

We identified two subthemes related to health concerns. First, worries about (the risk of) other conditions potentially linked to CD: respondents mainly worried about developing and managing deficiencies and their bone health. These worries were often related to uncertainty about their health because of a lack of support and follow-up. Second, health worries as a result of a late diagnosis: many respondents mentioned that it took years to get a diagnosis (for some >20 years), and they described how this increased their health concerns because they worried about the damage that was done in the meantime.

#### Inadequate treatment

Few people commented on this CD-QOL domain, but we did observe more variation in responses compared with other domains. Some respondents expressed the wish for better treatment options, whereas others were more positive about the diet and mentioned that it was a good thing that their condition can be controlled by something (relatively) simple as a diet.

#### CD-related restrictions

We identified three subthemes related to restrictions. First, lack of awareness or understanding of CD: respondents often struggled with the fact that others did not take their disease seriously. People felt frustrated that following a GFD is seen as a lifestyle choice rather than a necessity. Family and friends treat it as an inconvenience and believe they are being fussy out of ignorance. They also felt that restaurants see them as ‘awkward customers’ and often do not understand the concept of cross-contamination and how sick it can make people with CD.

Respondents also mentioned the lack of awareness among health professionals specifically. People felt that their symptoms were not taken seriously and/or were not recognised, which led to an unnecessarily late diagnosis. Some people had to ‘battle’ to finally get tested for CD, even when there was a family history of CD.

Second, restrictions of gluten-free products: although the choice and quality of gluten-free products have improved over the years, respondents were still generally unhappy with the limited availability of gluten-free products. Gluten-free alternatives are still more expensive, lack the taste associated with a particular food and are often less healthy (high in sugar). Additionally, it is hard to combine gluten free with other dietary requirements such as for type 1 diabetes, lactose intolerance or vegetarianism. People mentioned that they felt that the choice of products has reduced in favour of vegan foods, which have become the latest trend. Gluten-free prescriptions were helpful to cover the costs, but they were not available to everyone, and if they were, there was limited variety.

Third, restrictions on social life: CD and the strict nature of the diet impact respondents’ social lives, as everything needs a lot of planning, so doing anything spontaneously becomes very difficult. This is the case for eating out in restaurants, work events and eating at someone else’s place. This is especially difficult during holidays, and friends and family members can find the restrictions frustrating.

#### Lack of follow-up

Several people expressed concern regarding follow-up care after diagnosis, such as a lack of monitoring for secondary conditions, disease progression and treatment response. This was not captured by the CD-QOL questionnaire. There seemed to be large variation in the level of support and follow-up—some people were referred to a dietician and had regular check-ups with a gastroenterologist, whereas others were on their own after diagnosis.

#### Variation in QoL

Several possible causes for variation in QoL emerged from the free text analysis and are summarised with example quotes in table 3. Differences in when respondents were diagnosed can explain some of the variation in observed QoL. There is a period of adaptation to a gluten-free lifestyle in which the person with CD needs to learn how to follow a strict GFD, which is particularly difficult, but respondents mentioned that it gets easier with time. This suggests that the impact on QoL is particularly high immediately after diagnosis and decreases once people become accustomed to the diet. Our sample included people who had been diagnosed a long time ago and who were diagnosed recently, which could explain part of the variation we observed in QoL, which also fits with our finding that people who find the diet more difficult have lower QoL.

**Table 3:** Variation in quality of life

Cause for variation	Example quotes
Adaptation to a gluten-free lifestyle	Adaptation phase: *‘My whole way of life has changed on the back of my gluten-free diet; I have had to learn, and I am still learning to cook all over again. I cook very differently to how I used too which has taken time, food is a big part of family life now because everything has to be considered in advance.’* *‘I spend an inordinate amount of time reading food labels & have specific shops for specific products as each shop stock different things.’* Once adapted: *‘I think it can be a little overwhelming when you’re first diagnosed and realise what you can’t eat anymore, however I’ve found it reasonably easy to adapt and make all the family meals I did previously, I just make them all [gluten free*). *It just takes a little knowledge and planning.’* *‘It’s challenging, but with the right knowledge, support and research, it’s manageable.’*
Improvements in awareness and gluten-free products	*‘I was diagnosed more than 60 years ago as a toddler. The availability of gluten-free options was extremely limited then and is now transformed through much greater availability such that management of a gluten-free diet is easier. The notion of a gluten free diet is much more acceptable now and accommodated more readily in social situations than historically.’* *‘Things have got a lot easier since I was first diagnosed in 1963, age 3. I first had a biopsy in about 1987. The effect of 'gluten intolerance' increasing the commercial viability of GF foods has been tremendously helpful to making GF foods more available and of higher quality.’*
Symptom severity in response to gluten exposure	*‘I think CD is easy to manage once you understand what you can and can't have and what tolerance (if any) you have to cross contamination. I am very lucky that cross contamination doesn't cause me a big issue, but as a child I was incredibly sensitive and in pain a lot of the time.’*

Some people mentioned improvements in awareness and gluten-free products over time, which has made it easier to follow the diet. This was especially true for people who were diagnosed a long time ago and have experienced significant improvements. People who were diagnosed more recently and were still adapting to the diet were struggling with the lack of awareness. They compared gluten-free products to gluten-containing products instead of the first generation of gluten-free products.

Some of the QoL variability is likely related to the wide range in the severity of symptoms in response to gluten exposure. Some respond with acute and ‘embarrassing’ symptoms to minimal exposure, while others may be completely asymptomatic. This suggests that people who follow the diet very strictly may be people who get a strong response to minimal gluten exposure. These people are, therefore, more anxious about cross-contamination and feel more limited regarding eating out, which may explain why we observed lower QoL in people who follow the diet very strictly.

## Discussion

We found high variation in QoL among people with CD in the UK. This could partially be explained by variation in how difficult people found adhering to a GFD and how strictly people adhered to the diet. On average, people who found it more difficult and people who adhered very strictly had a lower QoL. The free text analysis suggested that people who adhere very strictly may do so because they experience symptoms with minimal gluten exposure. People who find it difficult to adhere to the diet may be people who only recently started the diet and are still adjusting to its impact.

The comments provided more context and additional sources of variation emerged. Some people experienced their disease and diet as a heavy burden and felt overwhelmed, whereas others found it manageable. Patients who worried about their health were concerned about the consequences of a late diagnosis, suggesting that people with a swift and straightforward diagnostic journey may be less affected by this. Some people felt very limited by the GFD and struggled to adjust to it, whereas others were quite positive about the diet and were happy that their symptoms could be managed by something relatively simple as a diet. In general, awareness and the availability and quality of gluten-free products have improved in recent years. Nevertheless, people struggled with the lack of awareness among friends and family, healthcare professionals and hospitality staff and were unsatisfied with the availability and costs of gluten-free products. The impact of a GFD on social life, causing social awkwardness and limiting spontaneity, was a burden to many. The comments also raised the issue that people with CD feel that follow-up care and support after diagnosis is lacking, which was not captured by the CD-QOL questionnaire.

Several studies have consistently shown that better adherence to a GFD is associated with improved QoL among individuals with CD.^15 16 22–25^ The degree of dietary adherence is variable, ranging between 42% and 91% across studies.^26^ Our study sample reported comparatively high levels of strict adherence to the diet (90%). Our sample showed an overall good QoL, comparable to studies in similar populations.^27 28^

A study conducted in the United States identified that “extreme vigilance” to a GFD resulted in significantly lower QoL scores.^29^ In their study, extreme vigilance and fear of gluten exposure led to increased anxiety and fatigue, lowering QoL as a result. Hypervigilance and fear of gluten exposure have also been linked to greater anxiety and depression.^30^ This finding fits with our observation that people with very strict adherence have lower QoL, although this may be confounded by symptom severity in response to gluten exposure.

In line with previous research, our study highlights the challenge of consistent follow-up care for individuals with CD. While regular follow-up is considered a vital component of patient care, in practice, follow-up care is often inconsistent or absent.^31 32^ Involving dieticians with relevant expertise can significantly benefit patient care by enhancing knowledge and minimising cross-contamination risks. However, an Australian survey of 5310 coeliac patients showed that over a third had not been seen by a dietician.^33^ Studies in the US and the UK have reported suboptimal follow-up rates, with a substantial portion of patients receiving irregular or no follow-up care.^34 35^

Our study further underscores the substantial treatment burden imposed by a strict and lifelong gluten-free diet. Patients with CD often perceive a high treatment burden compared with other common conditions.^36^ Anxiety and depression, which are common among individuals with CD, can further affect QoL and dietary adherence, emphasising the importance of psychological support.^30 33^ Finally, our findings align with existing evidence indicating that delays in the diagnosis of CD are associated with lower QoL.^13^

To our knowledge, this is the first study to use the CD-QOL tool in a UK population. The strength of this tool is that it is tailored to the unique experiences and challenges faced by individuals living with CD, enabling a more accurate assessment of the factors that influence their QoL. However, because the tool is specific to CD, the QoL scores cannot be compared directly to QoL in individuals without CD, such as other disease populations or the general population.

Although there was overall good agreement between the self-rated QoL and the CD-QOL scores, comparing the rates suggested that CD-QOL may underestimate QoL. People who rated their QoL as good had a ‘medium QoL’ on average according to the CD-QOL score. However, this does not affect the relationships we observed between QoL and diet adherence factors.

As our study design was cross-sectional, we are not able to tell the direction of the observed associations nor whether it is causal. For instance, people with a lower QoL may find it more difficult to adhere to the diet and people who struggle with adhering to the diet may have a lower QoL as a result. Although both may be true, the comments suggested that the latter is more likely, as people described how the limitations of the diet had a direct impact on their mental health and QoL.

The observed variation in QoL likely reflects the inherent heterogeneity of the sample and the disease itself. We did not collect data on a number of confounders of QoL, such as symptom severity or time since diagnosis, which is a limitation. Another limitation is that the study relies on self-reported outcomes, which may introduce biases and inaccuracies, particularly concerning dietary adherence. Although the study attempted to include participants from diverse deprivation levels and age groups, the sample used in this study is not representative of the broader UK population. There is an over-representation of highly educated white women from Southwest England as well as a higher proportion of respondents from more affluent neighbourhoods. The recruitment strategy may have introduced bias, as people who do not engage with online support groups will have been less likely to fill out the survey, including people with poor literacy, poor command of English, limited access to internet/computers and from more deprived areas. In addition, most respondents were from England, so results may not be generalisable to the whole of the UK. However, the QoL scores observed in this study were consistent with findings from other research.

The inclusion of a generic final comments box in the survey might have influenced the types of responses received. Participants may have been more inclined to leave comments when they had strong negative feelings or experiences to share, potentially leading to a higher frequency of negative comments compared with positive ones.

Healthcare professionals need to be educated about the importance of CD follow‐up and what best practice looks like. Our study has highlighted that guidance on diagnosis (such as offering tests to first-degree relatives with CD), follow-up (offering annual follow-up appointments and tests) and referral to a dietician are not always followed. Efforts should be made to ensure that access to healthcare is the same for everyone within the UK and does not depend on postcode, such as having access to gluten-free prescriptions. Our findings emphasise that increased support immediately following a coeliac diagnosis is needed, particularly aimed at helping patients adapt to a gluten-free lifestyle. This aligns with the recently published evidence-based monitoring guidelines, and concerted efforts should be made to ensure their implementation.

To better understand the factors involved in QoL in CD, future studies are needed in a large representative sample that collects information on potential confounding factors, including CD severity, level of disease control, age at and time since diagnosis, having relatives with CD, presence of other conditions and the level of support and follow-up received. The biases in the current study sample could be addressed by incorporating a multicentre project design, involving GP practices, community dieticians and hospitals across the country and the use of paper and online surveys and surveys in different languages. Subsequently, the data provided could serve as the foundation for developing a more comprehensive QoL instrument than the current CD-QOL. Future research should focus on finding the best approach to follow-up patients with CD, including which tests are needed and how often, since current guidelines on monitoring CD are largely based on expert opinion.^37^ Ultimately, new treatments are needed that can prevent damage from gluten, which would allow patients to be less strict, could be used when gluten has been ingested accidentally or would allow patients to eat a normal diet. However, until then, further research is also needed on how patients can be supported better immediately after diagnosis to help adjust to the GFD and minimise the impact on QoL.

## Conclusions

This study has highlighted the challenges faced by individuals with CD to adhere to a GFD in the UK and how this can affect their QoL. QoL may be improved by increased awareness as well as better availability of gluten-free options and gluten-free products on prescription and adequate support and follow-up care after diagnosis, which should be available for everyone, irrespective of location. Improved general awareness may increase the understanding of family and friends. Better education of hospitality staff is necessary to prevent accidental exposure to gluten, which would reduce anxiety and restrictions related to eating out.

## Data Availability

Data are available upon reasonable request. The datasets used and/or analysed during the current study are available from the corresponding author upon reasonable request.

## References

[1] Ludvigsson JF , Bai JC , Biagi F , et al . Diagnosis and management of adult coeliac disease: guidelines from the British Society of Gastroenterology. Gut 2014;63:1210–28. 10.1136/gutjnl-2013-306578 24917550 PMC4112432

[2] Rewers M . Epidemiology of celiac disease: what are the prevalence, incidence, and progression of celiac disease Gastroenterology 2005;128:S47–51. 10.1053/j.gastro.2005.02.030 15825126

[3] West J , Fleming KM , Tata LJ , et al . Incidence and prevalence of celiac disease and dermatitis herpetiformis in the UK over two decades: population-based study. Am J Gastroenterol 2014;109:757–68. 10.1038/ajg.2014.55 24667576 PMC4012300

[4] Al-Toma A , Volta U , Auricchio R , et al . European society for the study of Coeliac disease (ESsCD) guideline for coeliac disease and other gluten-related disorders. United European Gastroenterol J 2019;7:583–613. 10.1177/2050640619844125 PMC6545713 31210940

[5] Lebwohl B , Green PHR , Söderling J , et al . Association between celiac disease and mortality risk in a Swedish population. JAMA 2020;323:1277–85. 10.1001/jama.2020.1943 32259229 PMC7139272

[6] Lebwohl B , Granath F , Ekbom A , et al . Mucosal healing and risk for lymphoproliferative malignancy in celiac disease: a population-based cohort study. Ann Intern Med 2013;159:169–75. 10.7326/0003-4819-159-3-201308060-00006 23922062 PMC3788608

[7] Corazza GR , Di Stefano M , Mauriño E , et al . Bones in coeliac disease: diagnosis and treatment. Best Pract Res Clin Gastroenterol 2005;19:453–65. 10.1016/j.bpg.2005.01.002 15925849

[8] Parzanese I , Qehajaj D , Patrinicola F , et al . Celiac disease: from pathophysiology to treatment. World J Gastrointest Pathophysiol 2017;8:27–38. 10.4291/wjgp.v8.i2.27 28573065 PMC5437500

[9] Elwenspoek MM , Thom H , Sheppard AL , et al . Defining the optimum strategy for identifying adults and children with coeliac disease: systematic review and economic modelling. Health Technol Assess 2022;26:1–310. 10.3310/ZUCE8371 PMC9638887 36321689

[10] Gray AM , Papanicolas IN . Impact of symptoms on quality of life before and after diagnosis of coeliac disease: results from a UK population survey. BMC Health Serv Res 2010;10:105. 10.1186/1472-6963-10-105 20423498 PMC2907763

[11] Majsiak E , Choina M , Golicki D , et al . The impact of symptoms on quality of life before and after diagnosis of coeliac disease: the results from a Polish population survey and comparison with the results from the United Kingdom. BMC Gastroenterol 2021;21:99. 10.1186/s12876-021-01673-0 33663388 PMC7934494

[12] Murray JA , Watson T , Clearman B , et al . Effect of a gluten-free diet on gastrointestinal symptoms in celiac disease. Am J Clin Nutr 2004;79:669–73. 10.1093/ajcn/79.4.669 15051613

[13] Norström F , Lindholm L , Sandström O , et al . Delay to celiac disease diagnosis and its implications for health-related quality of life. BMC Gastroenterol 2011;11:118. 10.1186/1471-230X-11-118 22060243 PMC3233515

[14] Tontini GE , Rondonotti E , Saladino V , et al . Impact of gluten withdrawal on health-related quality of life in celiac subjects: an observational case-control study. Digestion 2010;82:221–8. 10.1159/000265549 20588037

[15] Usai P , Minerba L , Marini B , et al . Case control study on health-related quality of life in adult coeliac disease. Dig Liver Dis 2002;34:547–52. 10.1016/s1590-8658(02)80087-1 12502210

[16] Burger JPW , de Brouwer B , IntHout J , et al . Systematic review with meta-analysis: dietary adherence influences normalization of health-related quality of life in coeliac disease. Clin Nutr 2017;36:399–406. 10.1016/j.clnu.2016.04.021 27179800

[17] Fernández-Bañares F , Beltrán B , Salas A , et al . Persistent Villous atrophy in de novo adult patients with celiac disease and strict control of gluten-free diet adherence: a multicenter prospective study (CADER study). Am J Gastroenterol 2021;116:1036–43. 10.14309/ajg.0000000000001139 33491958

[18] Silvester JA , Comino I , Rigaux LN , et al . Exposure sources, amounts and time course of gluten ingestion and excretion in patients with coeliac disease on a gluten-free diet. Aliment Pharmacol Ther 2020;52:1469–79. 10.1111/apt.16075 32981131 PMC7780203

[19] Stefanolo JP , Tálamo M , Dodds S , et al . Real-world gluten exposure in patients with celiac disease on gluten-free diets, determined from Gliadin Immunogenic peptides in urine and fecal samples. Clin Gastroenterol Hepatol 2021;19:484–91. 10.1016/j.cgh.2020.03.038 32217152

[20] Rubio-Tapia A , Hill ID , Kelly CP , et al . ACG clinical guidelines: diagnosis and management of celiac disease. Am J Gastroenterol 2013;108:656–76. 10.1038/ajg.2013.79 23609613 PMC3706994

[21] Dorn SD , Hernandez L , Minaya MT , et al . The development and validation of a new coeliac disease quality of life survey (CD-QOL). Aliment Pharmacol Ther 2010;31:666–75. 10.1111/j.1365-2036.2009.04220.x 20015103

[22] Casellas F , Rodrigo L , Lucendo AJ , et al . Benefit on health-related quality of life of adherence to gluten-free diet in adult patients with celiac disease. Rev Esp Enferm Dig 2015;107:196–201. 25824917

[23] Häuser W , Stallmach A , Caspary WF , et al . Predictors of reduced health-related quality of life in adults with coeliac disease. Aliment Pharmacol Ther 2007;25:569–78. 10.1111/j.1365-2036.2006.03227.x 17305757

[24] Nachman F , del Campo MP , González A , et al . Long-term deterioration of quality of life in adult patients with celiac disease is associated with treatment noncompliance. Dig Liver Dis 2010;42:685–91. 10.1016/j.dld.2010.03.004 20399159

[25] White LE , Bannerman E , Gillett PM . Coeliac disease and the gluten-free diet: a review of the burdens; factors associated with adherence and impact on health-related quality of life, with specific focus on adolescence. J Hum Nutr Diet 2016;29:593–606. 10.1111/jhn.12375 27214084

[26] Hall NJ , Rubin G , Charnock A . Systematic review: adherence to a gluten-free diet in adult patients with coeliac disease. Aliment Pharmacol Ther 2009;30:315–30. 10.1111/j.1365-2036.2009.04053.x 19485977

[27] Siniscalchi M , Zingone F , Savarino EV , et al . COVID-19 pandemic perception in adults with celiac disease: an impulse to implement the use of Telemedicine. Dig Liver Dis 2020;52:1071–5. 10.1016/j.dld.2020.05.014 32425731 PMC7229921

[28] Marsilio I , Canova C , D’Odorico A , et al . Quality-of-life evaluation in coeliac patients on a gluten-free diet. Nutrients 2020;12:2981. 10.3390/nu12102981 33003417 PMC7601879

[29] Wolf RL , Lebwohl B , Lee AR , et al . Hypervigilance to a gluten-free diet and decreased quality of life in teenagers and adults with celiac disease. Dig Dis Sci 2018;63:1438–48. 10.1007/s10620-018-4936-4 29387990

[30] Ludvigsson JF , Lebwohl B , Chen Q , et al . Anxiety after coeliac disease diagnosis predicts mucosal healing: a population-based study. Aliment Pharmacol Ther 2018;48:1091–8. 10.1111/apt.14991 30288774

[31] Haines ML , Anderson RP , Gibson PR . Systematic review: the evidence base for long-term management of coeliac disease. Aliment Pharmacol Ther 2008;28:1042–66. 10.1111/j.1365-2036.2008.03820.x 18671779

[32] Pinto-Sanchez MI , Bai JC . Toward new paradigms in the follow up of adult patients with celiac disease on a gluten-free diet. Front Nutr 2019;6:153. 10.3389/fnut.2019.00153 31632977 PMC6781794

[33] Halmos EP , Deng M , Knowles SR , et al . Food knowledge and psychological state predict adherence to a gluten-free diet in a survey of 5310 Australians and New Zealanders with coeliac disease. Aliment Pharmacol Ther 2018;48:78–86. 10.1111/apt.14791 29733115

[34] Herman ML , Rubio-Tapia A , Lahr BD , et al . Patients with celiac disease are not followed up adequately. Clin Gastroenterol Hepatol 2012;10:893–9. 10.1016/j.cgh.2012.05.007 22610009 PMC3402703

[35] Bebb JR , Lawson A , Knight T , et al . Long-term follow-up of coeliac disease--what do coeliac patients want? Aliment Pharmacol Ther 2006;23:827–31. 10.1111/j.1365-2036.2006.02824.x 16556185

[36] Shah S , Akbari M , Vanga R , et al . Patient perception of treatment burden is high in celiac disease compared with other common conditions. Am J Gastroenterol 2014;109:1304–11. 10.1038/ajg.2014.29 24980880 PMC4159418

[37] Elli L , Leffler D , Cellier C , et al . Guidelines for best practices in monitoring established coeliac disease in adult patients. Nat Rev Gastroenterol Hepatol 2024;21:198–215. 10.1038/s41575-023-00872-2 38110546

