# Supplementary material for: Exploring factors influencing quality of life variability among individuals with coeliac disease: an online survey
Source: BMJ Open Gastroenterol. 2024 Jun 2;11(1):e001395. doi: 10.1136/bmjgast-2024-001395 (PMC11149133; doi:10.1136/bmjgast-2024-001395)
Supplement: Supplementary data [file bmjgast-2024-001395supp001.pdf]

Exploring factors influencing quality of life variability among individuals with coeliac disease: an online survey

Supplementary file

Authors

Martha M C Elwenspoek,[1,2] Jonathan Banks,[1,2] Prajakta Pratap Desale, Jessica Watson,[1,2] Penny Whiting.[2]

Affiliations

1. The National Institute for Health Research Applied Research Collaboration West (NIHR ARC West), University Hospitals Bristol NHS Foundation Trust, BS1 2NT Bristol, UK
2. Population Health Sciences, Bristol Medical School, University of Bristol, BS8 2PS Bristol, UK

Corresponding author

Martha M C Elwenspoek
9th Floor, Whitefriars, Lewins Mead,
Bristol, BS1 2NT
Email: Martha.Elwenspoek@bristol.ac.uk
Tel: +44/0 117 3427689

Elwenspoek M, *et al. BMJ Open Gastroenterol* 2024; 11:e001395. doi: 10.1136/bmjgast-2024-001395

Figure S1. Participant flow chart

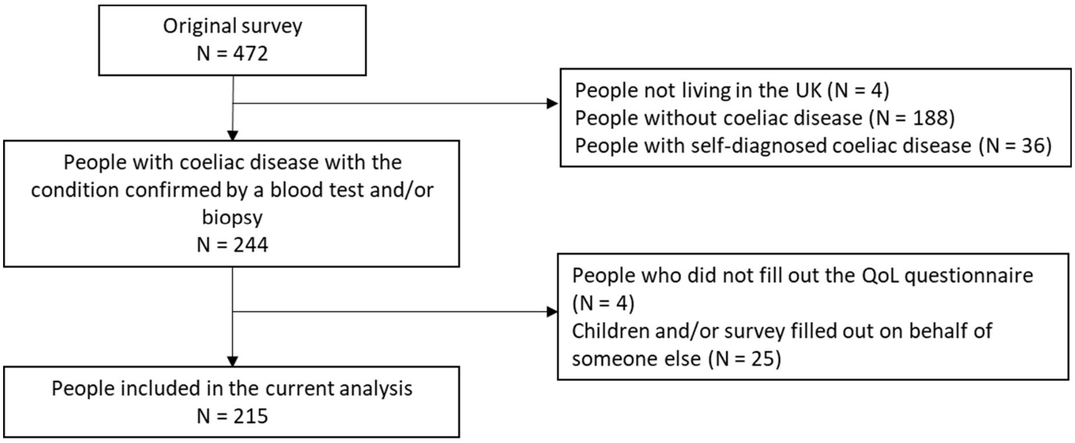

Elwenspoek M, *et al. BMJ Open Gastroenterol* 2024; 11:e001395. doi: 10.1136/bmjgast-2024-001395

Table S1. Gluten-free diet

	Total (N=215)	CD-QOL score Median (IQR)
Gluten free diet adherence*		
Not at all	3 (1.4%)	66.9 (50.3-83.4)
Not very strict	4 (1.9%)	49.4 (42.8-56.3)
Relatively strict	15 (7.0%)	77.5 (61.3-84.4)
Strict	148 (68.8%)	65.0 (46.3-77.5)
Super strict	45 (20.9%)	53.8 (35.6-62.5)
Gluten free diet difficulty		
Very easy	40 (18.6%)	78.8 (54.7-87.5)
Easy	75 (34.9%)	66.3 (55.0-77.5)
Neutral	39 (18.1%)	61.3 (51.2-69.4)
Difficult	55 (25.6%)	43.8 (33.8-55.6)
Very difficult	4 (1.9%)	26.3 (13.1-42.2)
Not applicable*	2 (0.9%)	66.9 (50.3-83.4)

* Diet adherence categories were described as follows: super strict, "I avoid all kinds of cross contamination. For instance, I do not eat out to avoid risk of gluten exposure"; strict, "I avoid cross contamination as much as possible. The only times I eat gluten is by accident (for instance when eating out)"; relatively strict, "I don't eat any foods containing gluten, but I'm not too fussy about cross contamination"; not very strict, "I sometimes make exceptions and eat something I know contains gluten"; not at all, "I try to lower my gluten intake but still eat gluten-containing products or I gave up on following the gluten-free diet".

Elwenspoek M, *et al. BMJ Open Gastroenterol* 2024; 11:e001395. doi: 10.1136/bmjgast-2024-001395

Table S2. Self-rated quality of life

	Total (N=215)	CD-QOL score Median (IQR)
Self-rated quality of life*		
Excellent	38 (17.7%)	81.3 (72.5-87.2)
Very good	71 (33.0%)	68.8 (56.9-78.1)
Good	76 (35.3%)	50.0 (39.7-61.3)
Fair	24 (11.2%)	36.3 (28.8-52.8)
Poor	6 (2.8%)	37.5 (35.8-53.8)

*"How would you rate your quality of life related to your illness?"
CD-QOL, coeliac disease quality of life; IQR, interquartile range.

Elwenspoek M, *et al. BMJ Open Gastroenterol* 2024; 11:e001395. doi: 10.1136/bmjgast-2024-001395

Table S3. Codes and all categorised comments

CD, coeliac disease; GF, gluten free; GP, general practitioner.

Name	Quote
**Dysphoria**	
Burdened or overwhelmed by CD	Hate not feeling well.<br><br>It took me 22 years to be diagnosed from when symptoms started and I worry about the damage that time has done. it also made it a lot harder to come to terms with.<br><br>It's just very hard to be happy when you're always worried about your health. and when you can't eat a Big Mac. god, I miss 3 junk food.<br><br>Having this disease is a heavy burden, the GF options are very expensive where they are available, choices are limited, you have to shop in 5 different supermarkets due to exclusivity of brands. It runs your life having to check ingredient labels constantly, being limited to eating out with friends and family. Feel persecuted and victimized at times it really upsets me when I can't be normal and included with everyone else.<br><br>Coeliac does impact on your life. Whilst GF is available some restaurants don't understand cross contamination and how sick it can make a person with this disease.<br><br>Since been diagnosed with coeliac disease food is all I think about, and where caters for gluten free.<br><br>I am on a strict GF diet, I can't eat gf oats or buckwheat or I get the same reaction as eating wheat (pain, cramps, blood in stools, mouth ulcers all over my tongue and inside my lips) brain fog , dry chapped skin and fatigue. It's the worst! I wouldn't wish on the worst person on earth!<br><br>It is very overwhelming and there are so many symptoms that can be related. There is limited wider understanding of the disease and it is often seen as an allergy, dietary choice or fad diet. I find it awkward to call and check with restaurants. Most of the time I feel okay with it, but I often have days when I feel down or frustrated.<br><br>I feel it's a very difficult disease and people don't realise how hard it is to deal with. How much you miss out on with family/ friends as I feel uncomfortable. They weight gain and just overall health implications of the disease such as having a B12 injection every three months for the rest of my life. Having to get blood tests done regularly. The amount of medication I had to take to get my levels back up to normal.<br><br>A GFD is a serious commitment to your lifestyle and as such should not be undertaken lightly.

	It's just tiresome, having to check in the supermarket, the labels all the time, just because it was ok last time, doesn't mean it is this time. Mostly it's expensive and always having to cook from scratch, is tedious.<br><br>It is incredibly overwhelming and impactful on my life now.<br><br>I live in constant fear of being contaminated when I eat out because it has happened frequently to me even though I am so careful, and there's not enough support for coeliacs dealing with this.
Insufficient support and information	I am still discovering things about my disease even now. I did not know there was a risk of infertility and higher risk of cancer until doing this survey. I am a female-presenting person of child-bearing age and not one doctor has mentioned that I might have trouble conceiving.<br><br>After diagnosis, I would prefer access to further health checks for osteoporosis, arthritis and other autoimmune conditions to be made more routinely and easily accessible. I often feel quite forgotten and alone when I try to discuss my worries about other symptoms which could be linked to Coeliac Disease.<br><br>I was just told I have coeliacs and to have a gf diet. And that was it. I feel that there should be more support and follow up tests to see if any other parts of your body are affected. My symptoms have improved a lot but I still don't feel well. I'm confident I do have coeliacs but think something else is also going on.<br><br>I wish there was more available information and that doctors looked at this before terrifying us with cancer tests and stats.<br><br>The diagnostic process should be made clear to all GPs and healthcare practitioners. I had a blood test and was told it looked like I could have coeliac disease. I was sent away to try a GF diet and only 4 months later when I visited the GP for something else was asked if I had had my endoscopy. I didn't know I needed one and was told to go back on a gluten-containing diet for at least 8 weeks. I refused because I had just started to feel a bit better. Was told I'd never be properly diagnosed and sent away from the GP as if it was my fault. I've since had to manage this on my own without any support.<br><br>There is definitely not enough information when you are first diagnosed and onwards over a ten year period I have become worse super super sensitive to gluten my reactions can be debilitating and I am also now lactose intolerant<br><br>Initially I found my diagnosis difficult, support from local GP was limited as they didn't seem to know too much specifically. My mother was diagnosed a few months before me after being extremely poorly for a long time, we actually found that we were given very different information on our diagnosis.<br><br>I have been given no information about what will happen next/ if my son might need to get checked out. I find being a coeliac without symptoms quite challenging.<br><br>There needs to be better education for the blood relatives of coeliacs so they know all about the risks of not being diagnosed.
Social anxiety	I really hope that there is a solution to cross contamination and, if accidently occurs then there should be a medicine to prevent

Elwenspoek M, *et al. BMJ Open Gastroenterol* 2024; 11:e001395. doi: 10.1136/bmjgast-2024-001395

	damage. As liven gluten free isn’t impossible. However, avoiding all gluten even at microscopic levels is very stressful and, it makes life a lot harder.<br><br>when eating out or having food prepared by someone else, I feel that there is a stigma around eating GF and many think that it's a health choice and it is not taken seriously enough (especially by those in the restaurant business). That then makes the diet more difficult to follow as eating out is such a big part of everyone's social life but is also a massive risk.<br><br>I also feel awkward socially as I feel like I'm being a pain having to check everything and other people might think I'm being difficult as they don't necessarily understand why it's a problem. I also worry about accidental contamination and having a reaction while I'm out (which has happened a few times) especially if I'm far from home as it's usually quite immediate/severe and would be especially embarrassing if it was with people I didn't know or a work thing for example.<br><br>I feel an inconvenience to family occasions and celebrations & often opt not to eat rather than come across as problematic. This disease begins to affect your personality & your mental health as much as your physical health.<br><br>I find it awkward to call and check with restaurants.<br><br>How much you miss out on with family/ friends as I feel uncomfortable.<br><br>Hate being the 'fussy' one (which I'm not!!) when eating out with company.<br><br>I am less anxious about the effects of the disease because I am following the diet carefully. The danger of cross contamination is by far the biggest ‘headache’ for my daily life as I live and work with several people who eat gluten. We all - especially I - need to have constant vigilance about crumbs etc. This can be mentally tiring. I am anxious that one day someone will make a serious mistake.<br><br>More than feeling it restricts socialising it is the awkwardness of choosing places to eat. This is especially the case when travelling abroad and whilst I have done plenty of holidays and some travelling, I would worry about it travelling on some continents but hope it won't deter me in the future.<br><br>I am personally quite lucky as I don't have many of the typical symptoms, and the symptoms I do have are relatively mild compared to some, but it can be very difficult to accept that what you're eating is safe to eat if you haven't made it yourself.<br><br>I live in constant fear of being contaminated when I eat out because it has happened frequently to me even though I am so careful
**Health concerns**	
Worried about (risk of) secondary conditions	I found out that I have osteoporosis at 29 years old and even though there's a slim chance I could rebuild some bone strength (because I’m so young) I have only been given the standard bone health help for elderly people and no tailored advice for someone young who is actively trying to BUILD bone. it's just very hard to be happy when you're always worried about your health.<br><br>After diagnosis, I would prefer access to further health checks for osteoporosis, arthritis and other autoimmune conditions to be made more routinely and easily accessible. I often feel quite forgotten and alone when I try to discuss my worries about other

Elwenspoek M, *et al. BMJ Open Gastroenterol* 2024; 11:e001395. doi: 10.1136/bmjgast-2024-001395

	symptoms which could be linked to Coeliac Disease.<br><br>The other ongoing issue is ensuring I get sufficient nutrients. I've been Vit D deficient a number of times & am currently B12 deficient & attempting to correct via diet otherwise I'll have to have injections.<br><br>my bones have been affected as after s lift accident l now have bone spurs right by my spinal cord and worry l could become paralysed. I was fit and l thought healthy until the lift dropped and stopped suddenly causing issues with my already compromised bones.<br><br>I feel like not enough information is out there about CD, and that more than bloating, constipation, nausea, I got diagnosed because I was having miscarriages, and now sadly I am infertile. I have low b12 and iron count, my hair falls out, my bones are weaker, and my small intestine has still not recovered over the last 8 years.<br><br>I feel it's a very difficult disease and people don't realise how hard it is to deal with. How much you miss out on with family/ friends as I feel uncomfortable. They weight gain and just overall health implications of the disease such as having a B12 injection every three months for the rest of my life. Having to get blood tests done regularly. The amount of medication I had to take to get my levels back up to normal.<br><br>I was sent for a bone density scan a few months later and it was discovered I also have osteopenia. I would like to know more about how long I may have unknowingly had the disease. It is also frustrating that I have no clear way of knowing whether the lining of my gut is healing/healed.<br><br>After a series of blood tests showed low ferritin levels despite a healthy diet, I now take iron daily and expect to do so for life. I am also prescribed calcium with vitamin D3 due to osteopenia found on a routine DEXA scan. Seven weeks ago, I had a total hip replacement and have been told that part of the joint had worn away, possibly due to having osteopenia which is a worry for me.<br><br>My GP was excellent, but I am worried about the lack of regular check-ups for deficiencies etc.<br><br>I now have pernicious anaemia, caused by my coeliac disease
Worries about late diagnosis	I wasn't given enough information about coeliac disease or how the diagnosis process worked when I was going through diagnosis. It also took a few years before I saw a GP who considered coeliac disease despite me being very symptomatic. I think racism and ideas about me being hypochondriac and/or mentally ill prevented GP's from testing me. This led to a further 10 years of my health deteriorating and what now seems to be permanent damage to my body. I have ME/CFS, fibromyalgia and dermatitis herpetiformis. I think I might not have developed these conditions if I'd been diagnosed much earlier.<br><br>it took me 22 years to be diagnosed from when symptoms started, and I worry about the damage that time has done. it also made it a lot harder to come to terms with.<br><br>I wasn't diagnosed until I was in my forties! My first child has cerebral palsy and there's never been a reason why? I often wonder if my Coeliac disease could have caused this? Especially in the early development stage of the embryo?

Elwenspoek M, *et al. BMJ Open Gastroenterol* 2024; 11:e001395. doi: 10.1136/bmjgast-2024-001395

	I was 42 when I was finally diagnosed. I started visiting the doctor with symptoms, mainly, extreme fatigue when I was about 15. It took 27 years to get a diagnosis! I really worry about the damage that was done in that time to my long-term health. I don't think doctors think about coeliac disease, they immediately think you have anaemia and give you iron. I wish I had been diagnosed earlier!<br><br>I should have been diagnosed decades ago but my GP said I had IBS. Now I am on treatment for osteoporosis and left feeling I might be at risk of various cancers.<br><br>I have concerns of the long-term effect on health since I had Coeliac disease probably since my late teens & was diagnosed at 46 years old.<br><br>I found it quite difficult to get diagnosed with coeliac disease as it was the last thing that the doctors looked for, I didn’t have traditions symptoms except for weight loss and it was only when I caught pneumonia and almost died, needed a blood transfusion that more investigation was done. I had to push for it though, I was told I was anaemic but not given any reason why, just that it happens, I had to insist on more investigations for that and was really very ill for a long time. I’m still not 100% and am told I may never be now.
**Inadequate treatment**	
Wish for better treatments	In one way it's good that it can be controlled by a strict diet but it would be great to have a medication that means you can eat gluten, more for the social aspect of being able to be spontaneous when eating out or where to go on holiday, instead of having poor choices of restaurant is due to where you live, and having to plan in advance or go self-catering.<br><br>I really hope that there is a solution to cross contamination and, if accidently occurs then there should be a medicine to prevent damage. As liven gluten free isn’t impossible. However, avoiding all gluten even at microscopic levels is very stressful and, it makes life a lot harder.<br><br>I do wish there were alternative treatments & I know there was some hope with a vaccine style injection a couple of years back.<br><br>I also wish there was another treatment other than a gf diet, because I really miss bread and hate being the 'fussy' one (which I'm not!!) when eating out with company.
**CD related restrictions**	
Lack of awareness, understanding	Generic, general public<br>I also think that people do not realise the seriousness of the disease and the other diseases it can cause.<br><br>For me, having coeliac disease has affected travelling, socialising, and eating out quite a lot. I find eating out difficult and I can't see that there is much incentive (financial or otherwise) for restaurants to make this easier/safer or provide more options for coeliacs. I also feel awkward socially as I feel like I'm being a pain having to check everything and other people might think I'm being difficult as they don't necessarily understand why it's a problem.<br><br>I feel like not enough information is out there about CD, and that more than bloating, constipation, nausea, I got diagnosed because

	I was having miscarriages, and now sadly I am infertile.<br><br>There is limited wider understanding of the disease and it is often seen as an allergy, dietary choice or fad diet.<br><br>people don't realise how hard it is to deal with<br><br>I think the wider general public still don't realise it's a disease rather than a diet fad/choice. It seems to be treated as a major inconvenience rather than veganism which has been embraced by shops/restaurants. I really don't think shops/chefs realise how little they have to change sometimes.to make something gluten free.<br><br>Feel it is a little-known disease and became "trendy" for a while when people found not eating bread could help with bloating, think they self-diagnosed allergies which detracted from genuine Coeliac suffers<br><br>I strongly believe that people need to know more about the prevalence and severity of the condition.<br><br>There is continual frustration with the common misconception that it is a lifestyle choice not a disease that can have horrid symptoms.<br><br>Seen as a fad, not truly understood by people. Level of knowledge in restaurants etc is not good.<br><br>I hope for the medical understanding to be circulated into the greater population and commercial entities for better understanding, better practice in hospitality settings and for food manufacturers/supermarkets as the products are restricted and some big supermarkets putting food choice (vegan/vegetarian options) above dietary needs.<br><br>I would also like to see better public education regarding the Gluten Free diet so that it is not promoted as health for those without CD or gluten intolerance.<br><br>There needs to be greater awareness and education within the food industry, schools, and the workplace to prevent bullying, ignorance and mistakes.<br><br>Family and friends<br>I believe that everyone should take some understanding of coeliac disease and the impact it causes a person. I was diagnosed early 20's and from myself being tested positive and living a gluten free life. My parents are now being tested but my siblings believe coeliac disease is nothing to worry about and that you should still be able eat whatever with no issue.<br><br>I was unhappy that I had the symptoms you described for several months, and was told it was stress and I needed to relax. Only when my ankles swelled was I given blood tests, and the CD discovered.<br><br>Think it's generally misunderstood- family and friends thinking I'm making a fuss.<br><br>Healthcare professionals

Elwenspoek M, *et al. BMJ Open Gastroenterol* 2024; 11:e001395. doi: 10.1136/bmjgast-2024-001395

	Doctors don't know enough about the other symptoms only most common.<br><br>Not all symptoms are obvious, I think there needs more awareness about that. As damage can be done long term.<br><br>You can never expect GPs to know everything but having had to battle with GPs for a year for one daughter when she was not growing before being tested and then having another who is so reactive cannot be confirmed as they insist she has to be on gluten and do the blood tests... but she has been that way since weaning - I have one confirmed daughter and 1 that is down as being treated as though confirmed coeliac but they cannot actually confirm, as stated above...<br><br>Think more training on understanding CD for health professionals - I spent over 15 years waiting to be diagnosed, despite being under the care of 2 consultant gastroenterologists during that time.<br><br>Length of time to be diagnosed. I was diagnosed anaemic six years ago but not offered coeliac test. I was only diagnosed recently following further symptoms and cutting out gluten myself. I went back on gluten for 6 weeks for the test. I feel doctors should be more knowledgeable and ready to test for it.<br><br>The diagnostic process should be made clear to all GPs and healthcare practitioners. I had a blood test and was told it looked like I could have coeliac disease. I was sent away to try a GF diet and only 4 months later when I visited the GP for something else was asked if I had had my endoscopy. I didn't know I needed one and was told to go back on a gluten-containing diet for at least 8 weeks. I refused because I had just started to feel a bit better. Was told I'd never be properly diagnosed and sent away from the GP as if it was my fault. I've since had to manage this on my own without any support.<br><br>GPs don't know enough about it. Don't know the symptoms, eating gluten free for 6 weeks. Was fobbed off so many times that my symptoms were because of a stressful job. Diagnosed with anaemia before Coeliac Disease but no follow up blood test to see the tablets weren't being absorbed. I had to ask for a blood test.<br><br>It took 11 years to obtain the correct diagnosis - most of that time my symptoms were dismissed as irrelevant.<br><br>I would also add that the process of getting diagnosed with coeliac disease was pretty tough, with some doctors all but dismissing me and my symptoms. I also think awareness should be raised about symptoms in other medical professions eg my first symptom was enamel erosion on my teeth which came before anything else. I spoke to a number of dentists about this with none of them having any idea about the cause - I am not sure how common that symptom is but could be a 3 way to identify the disease in more people.<br><br>Restaurants<br>Whilst GF is available some restaurants don't understand cross contamination and how sick it can make a person with this disease.<br><br>It's easy to sick to the GF diet when preparing food for yourself. However, when eating out or having food prepared by someone else, I feel that there is a stigma around eating GF and many think that it's a health choice and it is not taken seriously enough (especially by those in the restaurant business). That then makes the diet more difficult to follow as eating out is such a big part of everyone's social life but is also a massive risk.

	I think having more choice of food would make a great impact and educating restaurants/cafes so we aren't just branded as 'awkward customers' would be great.<br><br>The increase in vegan foods has limited the GF options, and restaurants often get confused between dairy free and GF (I've been given dairy free food in the past which has made me ill)<br><br>Supermarkets<br>I wish non coeliac people understood that this is not a fad diet like veganism. Supermarket's pander to the latest fads and I feel my choices in food are reduced as a result.
Effect on social life	The social aspect of being able to be spontaneous when eating out or where to go on holiday, instead of having poor choices of restaurant is due to where you live, and having to plan in advance or go self-catering.<br><br>I miss just being able to eat where I want to. Not feel left out of work outings.<br><br>It runs your life having to check ingredient labels constantly, being limited to eating out with friends and family.<br><br>when eating out or having food prepared by someone else, I feel that there is a stigma around eating GF and many think that it's a health choice and it is not taken seriously enough (especially by those in the restaurant business). That then makes the diet more difficult to follow as eating out is such a big part of everyone's social life but is also a massive risk.<br><br>For me, having coeliac disease has affected travelling, socialising, and eating out quite a lot. I find eating out difficult and I can't see that there is much incentive (financial or otherwise) for restaurants to make this easier/safer or provide more options for coeliacs. I also feel awkward socially as I feel like I'm being a pain having to check everything and other people might think I'm being difficult as they don't necessarily understand why it's a problem.<br><br>I feel that although the GF diet is easier to follow now than when I was diagnosed (6 years ago) it is still a horrible choice to have. I feel for children diagnosed as they must struggle with not being allowed treats and parties must be difficult.<br><br>I miss being able to be spontaneous & just randomly go out for lunch opposed to planning everything.<br><br>Needing to follow gf diet has curtailed my spontaneity in travelling & eating out. I need to research a lot before going & ask lots before deciding to eat somewhere.<br><br>Holidays are very difficult.<br><br>Food out and about is always tricky, and I know my family find it frustrating when we are on holiday etc, plus limited options.<br><br>Also, it's a choice of being a fussy pants with food whenever you go out or go to see friends or staying at home and not seeing

Elwenspoek M, *et al. BMJ Open Gastroenterol* 2024; 11:e001395. doi: 10.1136/bmjgast-2024-001395

	anyone. I choose the former. But you can't expect friends and restaurants to always 'get it' in terms of not feeding you gluten. It's not your friends' fault. There's no way someone would know how to cut out gluten 100% if they don't have to themselves. I don't want to be a fussy pants so I always take a bag of food with me everywhere I go.<br><br>The most restrictive things are: Eating on the go, Eating out, Cost of staples such as bread
GF products	
Availability	I know since I was diagnosed that has improved a lot.<br><br>Very limited GF food available in hospital, nothing at breakfast time.<br><br>choices are limited, you have to shop in 5 different supermarkets due to exclusivity of brands.<br><br>I am now a pensioner. l am struggling to buy food that l can eat. meat veg and etc are fine but as l only get my bread on prescription my diet is not as varied as l would like it to be. l pay out a lot of money in supplementation to keep me healthy and the right weight<br><br>Supermarket's pander to the latest fads and I feel my choices in food are reduced as a result.<br><br>It's often said now that there's more choice of gf food in supermarkets, but provision is patchy. You can't rely on going shopping and getting even the basics at your local supermarket. Also, gf food on prescription is fortified with iron and calcium and so supports good health in coeliacs, but it's not available from all health authorities. Supermarket gf food is often high in sugar; reading food labels becomes a useful habit.<br><br>GF food should be cheaper and more accessible.<br><br>I think having more choice of food would make a great impact and educating restaurants/cafes so we aren't just branded as 'awkward customers' would be great.<br><br>availability of gluten free options is annoying as is the price.
Costs	GF options are very expensive.<br><br>I am now a pensioner. l am struggling to buy food that l can eat. meat veg and etc are fine but as l only get my bread on prescription my diet is not as varied as l would like it to be. l pay out a lot of money in supplementation to keep me healthy and the right weight<br><br>GF food should be cheaper and more accessible.<br><br>The price of food which is limited at best is not fair and the size difference compared to regular foods is shocking sometimes it is literally half the size for twice the price.<br><br>The cost of following a gluten free diet

	The cost of essential GF is extortionate. Flour, bread, pastry. I n Bedfordshire I do not get gluten free essential products on prescription in the last 7 years, and this has had an impact on what I bake from scratch at home as flour and bread is do expensive to buy.<br><br>Availability of gluten free options is annoying as is the price.<br><br>There is a lot more food available now, but staple foods should be on prescription, it is too expensive at the moment.<br><br>The challenge of obtaining even basic gluten free items on prescription now also impacts on affordability of a gluten free diet with comparable products being considerably more expensive and this will inevitably impact on levels of compliance where financial constraints apply to an individual/family.<br><br>I think there should be more financial support towards the cost of eating GF for coeliacs the cost of the food has impacted my shopping budget a lot. I worry the financial impact it would have if my 4 children also had the disease.<br><br>Access to reasonably priced GF food for those on low incomes needs to be addressed, as it's becoming more difficult to access on prescription.<br><br>A GF diet is expensive, I am fortunate that I can afford it.<br><br>It is really frustrating that they cost so much more than gluten equivalent.<br><br>The most restrictive things are: Eating on the go, Eating out, Cost of staples such as bread
Other dietary requirements	I wish there were a lot more gluten free vegetarian foods although it has got a lot better recently.<br><br>As a type 1 diabetic I was diagnosed before I had any symptoms which made changing to the diet even harder & in the first 12 months, I wasn’t very strict.<br><br>Just to say that my own dieting experience has been rocked further since being diagnosed with diabetes and switching to a low car diet - this rules out most 'free from' type products!<br><br>Gluten free diet is okay - was amazing whilst fashionable but recently vegan food has become fashionable, and a lot of food has seen wheat flour creeping back as a result and now that with Brexit and covid affecting so many companies a lot of factories have altered and there are more foods with risk of contamination too... so choices are gradually limiting...but we are still lucky and prices for many things have dropped so much.<br><br>availability of gluten free options is annoying as is the price. Also, it seems to be sacrificed in shops for space for vegan food at present.<br><br>The increase in vegan foods has limited the GF options, and restaurants often get confused between dairy free and GF (I’ve been

Elwenspoek M, et al. BMJ Open Gastroenterol 2024; 11:e001395. doi: 10.1136/bmjgast-2024-001395

	given dairy free food in the past which has made me ill)<br><br>January is the hardest when shops include a lot of vegan products often at the expense of their free from range. My personal frustration is that gluten free foods will 9 times out of 10 will also include dairy free and other free from a together. It's hard when you do not have a dairy allergy or other allergies that are lumped together.<br><br>It is harder being vegetarian as well as many vegetarian processed foods are not gluten free eg Most Linda McCartney products, Quorn products as they often have wheat or barley malt. I wish they could change this.
Positives	
Taste	Gluten free food not good not taste bland.<br><br>I find eating simple & plain foods is better than eating the GF foods.<br><br>GF food tries to copy ordinary food, but the taste is never as good although you do get used to it
**Lack of follow up**	
Lack of follow up	After diagnosis, I would prefer access to further health checks for osteoporosis, arthritis and other autoimmune conditions to be made more routinely and easily accessible. I often feel quite forgotten and alone when I try to discuss my worries about other symptoms which could be linked to Coeliac Disease.<br><br>I was just told I have coeliacs and to have a gf diet. And that was it. I feel that there should be more support and follow up tests to see if any other parts of your body are affected. My symptoms have improved a lot, but I still don't feel well. I'm confident I do have coeliacs but think something else is also going on.<br><br>I was sent for a bone density scan a few months later and it was discovered I also have osteopenia. I would like to know more about how long I may have unknowingly had the disease. It is also frustrating that I have no clear way of knowing whether the lining of my gut is healing/healed.<br><br>It has historically taken a relatively long time to be officially diagnosed but the benefit of such a diagnosis (via blood test and biopsy) is getting staple food on prescription (in some regions) and having annual blood tests to check for deficiencies due to CD. Some health authorities also offer annual check-ups with a gastro specialist and/or dietitian. But it seems to be a postcode lottery what support you're offered after diagnosis!<br><br>My follow-on care has been lacking, I've been diagnosed within the last 6 months and have had a follow up telephone appointment and then pretty much signed off. Told to speak to my GP in a year, no information about whether bloods are needed to confirm I'm on the right track etc<br><br>It took me 25 years to finally get a diagnosis. Awful and now follow ups don't happen unless I ask.<br><br>The diagnostic process should be made clear to all GPs and healthcare practitioners. I had a blood test and was told it looked like I could have coeliac disease. I was sent away to try a GF diet and only 4 months later when I visited the GP for something else was

	asked if I had had my endoscopy. I didn't know I needed one and was told to go back on a gluten-containing diet for at least 8 weeks. I refused because I had just started to feel a bit better. Was told I'd never be properly diagnosed and sent away from the GP as if it was my fault. I've since had to manage this on my own without any support.<br><br>My GP was excellent, but I am worried about the lack of regular check-ups for deficiencies etc.<br><br>Follow up appointments with specialist have not been very helpful, no referral to a dietician for example.<br><br>As a celiac, especially one who was diagnosed based on skin symptoms and not on GI symptoms, I wish there was a way/system in place where doctors regularly checked your levels of gluten Abs or the damage to your villi to see if the GF diet, as the patient is adhering to it, is working as an effective treatment. Additionally, I feel that screening for known risk factors as a result of being a celiac doesn't exist. My doctor knows I am a celiac (and as such I get offered the flu jab) but there is nothing else done to further screen for the other issues (cancer, bone problems, etc.) I am at risk for, or even to discuss what I should be looking out for in my day-to-day life.
**Potential causes for variation**	
Adapting to GF lifestyle	I spend an inordinate amount of time reading food labels & have specific shops for specific products as each shop stock different things.<br><br>I am eating gluten free for almost 12 months and the difference it has made to my life yes it takes a bit of time to get used to it but I never want to get as sick as I was so I will not be going for biopsy as Gluten free diet has changed my life.<br><br>It's challenging but with the right knowledge, support, and research it's manageable. It does heighten anxiety when thinking of 'returning to normal' having been diagnosed whilst in the pandemic I've found it easier to adjust to.<br><br>Coeliac disease diagnosis made me get start cooking from scratch again. I miss all the things I can't have but in 12 months I have adjusted to my new diet and know it's for the best treatment for my long-term health.<br><br>Having had this condition most of my life, it has become the "normal".<br><br>My whole way of life has changed on the back of my gluten free diet, I have had to learn, and I am still learning to cook all over again. I cook very differently to how I used too which has taken time, food is a big part of family life now because everything has to be considered in advance.<br><br>I'm newly diagnosed so still trying to adapt.<br><br>I think it can be a little overwhelming when you're first diagnosed and realise what you can't eat anymore, however I've found it reasonably easy to adapt and make all the family meals I did previously, I just make them all gf. It just takes a little knowledge and planning.<br><br>Cutting out gluten isn't easy to start with, you have no idea it's in everything until you have to cut it out. But it gets easier.

	I was diagnosed aged 11, in 1997/8, into a family full of coeliacs. As a result, to me following a gf diet is now second nature, and so I'm aware my opinions might be quite different to a newly diagnosed patient with no previous awareness of the disease.<br><br>I think coeliac disease is easy to manage once you understand what you can and can't have and what tolerance (if any) you have to cross contamination. I am very lucky that cross contamination doesn't cause me a big issue, but as a child I was incredibly sensitive and in pain a lot of the time.
Improved awareness and GF products	The diet is now much easier to follow because supermarkets stock gluten free products and restaurants are more aware of the issues.<br><br>In the 12 years since diagnosis the general understanding and availability of products has improved hugely.<br><br>I was diagnosed more than 60 years ago as a toddler. The availability of gluten free options was extremely limited then and is now transformed through much greater availability such that management of a gluten free diet is easier. The notion of a gluten free diet is much more acceptable now and accommodated more readily in social situations than historically.<br><br>Things have got a lot easier since I was first diagnosed in 1963, age 3. I first had a biopsy in about 1987. The effect of 'gluten intolerance' increasing the commercial viability of GF foods has been tremendously helpful to making GF foods more available and of higher quality.<br><br>It is a restricting diet but there are many more products available in supermarkets these days.<br><br>Gluten free options are so much easier to come by these days, however, it is really frustrating that they cost so much more than gluten equivalent.
Positive attitudes toward GF diet and having coeliac disease	It's easy to sick to the GF diet when preparing food for yourself.<br><br>There is a lot of support on social media. The coeliac UK website helped me in the early months of diagnosis.<br><br>Although it does have its restrictions, it is an easy diet to follow. Just cook from fresh so you know what is in your food...! Batch prep is good too!<br><br>Not as daunting as I first thought.<br><br>Being coeliac is not the worst thing that has happened to me. The relief when I was diagnosed 4/5 years ago was great. The c. 5 years of getting thinner and vomiting etc before that was not fun.<br><br>There are many greater choices in food and eating out than when first diagnosed. And I live in an area where shops do stock a decent range of GF foods.

Elwenspoek M, *et al. BMJ Open Gastroenterol* 2024; 11:e001395. doi: 10.1136/bmjgast-2024-001395

	I think the gluten free diet is a very small price to pay for the benefits on the health of anyone with coeliac disease.<br><br>My quality of life is good and eating a gluten free diet is not a problem for me.<br><br>I feel so much better on the gluten free diet. There's no question of me eating gluten again, going back to my old way of eating. If I have to have a serious condition, I'm so glad it's coeliac rather than something else. So lucky to be able to deal with it through diet and that's the end of it.<br><br>Coeliac disease is as limiting as you make it. It is easy to cope with and lead a normal life, although preplanning is sometimes needed.<br><br>It sucks, but it's better than having one of the inflammatory bowel diseases.<br><br>Although coeliac disease is a serious autoimmune disease, I can think of far worse illnesses to have. If only every serious illness could be fixed as simply as cutting out gluten.
Positive comment on follow up care	Hospital care outstanding, checks (bone scans, bloods etc..) all given within a year of diagnosis. Yearly bloods checked, very understanding staff and a phone number I can ring to speak to the specialist nurse for the rest of my life. Very impressed!

Elwenspoek M, *et al. BMJ Open Gastroenterol* 2024; 11:e001395. doi: 10.1136/bmjgast-2024-001395
